# Predicting healthcare expenditure based on Adjusted Morbidity Groups to implement a needs-based capitation financing system

**DOI:** 10.1186/s13561-024-00508-4

**Published:** 2024-05-08

**Authors:** Jorge-Eduardo Martínez-Pérez, Juan-Antonio Quesada-Torres, Eduardo Martínez-Gabaldón

**Affiliations:** 1 Department of Applied Economics, University of Murcia, Campus of Espinardo, Murcia, 30100 Spain; 2 Department of Health of the Region of Murcia, 4 Pinares Street, Murcia, 30001 Spain; 3 International Doctorate School of the University of Murcia (EIDUM), PhD Program in Economics (DEcIDE), Murcia, Spain; 4 Department of Financial Economics and Accounting. University of Alicante, Carrer San Vicente de Raspeig, Alicante, 03690 Spain

**Keywords:** Healthcare expenditure, Capitation financing model, Adjusted Morbidity Groups

## Abstract

**Background:**

Due to population aging, healthcare expenditure is projected to increase substantially in developed countries like Spain. However, prior research indicates that health status, not merely age, is a key driver of healthcare costs. This study analyzed data from over 1.25 million residents of Spain's Murcia region to develop a capitation-based healthcare financing model incorporating health status via Adjusted Morbidity Groups (AMGs). The goal was to simulate an equitable area-based healthcare budget allocation reflecting population needs.

**Methods:**

Using 2017 data on residents' age, sex, AMG designation, and individual healthcare costs, generalized linear models were built to predict healthcare expenditure based on health status indicators. Multiple link functions and distribution families were tested, with model selection guided by information criteria, residual analysis, and goodness-of-fit statistics. The selected model was used to estimate adjusted populations and simulate capitated budgets for the 9 healthcare districts in Murcia.

**Results:**

The gamma distribution with logarithmic link function provided the best model fit. Comparisons of predicted and actual average costs revealed underfunded and overfunded areas within Murcia. If implemented, the capitation model would decrease funding for most districts (up to 15.5%) while increasing it for two high-need areas, emphasizing allocation based on health status and standardized utilization rather than historical spending alone.

**Conclusions:**

AMG-based capitated budgeting could improve equity in healthcare financing across regions in Spain. By explicitly incorporating multimorbidity burden into allocation formulas, resources can be reallocated towards areas with poorer overall population health. Further policy analysis and adjustment is needed before full-scale implementation of such need-based global budgets.

**Supplementary Information:**

The online version contains supplementary material available at 10.1186/s13561-024-00508-4.

## Introduction

The expected evolution of public spending in developed countries, particularly in healthcare services due to population aging, presents a significant challenge [1–3]. Concerns about the impact on healthcare expenditure are reinforced by empirical evidence showing a direct relationship between age and average per capita expenditure. Indeed, the commonly observed "J-curve" profile in other countries [4–7] has also been frequently described for Spain [8–12]. However, there is increasing evidence indicating that this relationship between age and healthcare expenditure is greatly attenuated when considering the population's health status [12–14]. In fact, a significant portion of healthcare spending in developed countries is concentrated among population groups with high healthcare needs derived from their health status [15], with evidence supporting this in nearly all developed countries [16–18]. In recent years, there has been a growing interest in the instruments used to measure multimorbidity and their ability to explain relevant clinical and economic outcomes [19]. Therefore, different healthcare financing models should not overlook this reality, and it is precisely in this context that the emergence of population stratifiers is justified. These stratifiers allow us to go beyond purely demographic variables such as age and sex and consider the population's health status, particularly regarding chronic health problems and multimorbidity.

Traditional healthcare models of financing include salary, fee-for-service (FFS), Diagnosis-Related Group (DRG) and capitation [20, 21]—it is the latter, the capitation financing models, aiming to improve care and financial outcomes with a comprehensive perspective beyond hospital centers, considering population care as an integral aspect that necessarily encompasses all levels of care. It is in the analysis and application of these financing models where population stratification tools emerge, primarily focusing on demographic and health variables. The expected financing for a specific management area would be based on its adjusted or equivalent population, which would be the result of applying a coefficient to its natural population based on the characteristics considered in the analysis (demographic, health, socioeconomic, etc.).

Therefore, the objective of this study is to contribute to the existing literature on healthcare financing models by simulating an area-based budgeting model as a result of applying a pure capitation-based model within the Murcian Health Service, using population stratification by AMGs, age, and sex, along with patient cost information extracted from the Murcian Health Service's analytical accounting system. This analytical accounting system provides cost per patient information by incorporating all costs that each patient directly or indirectly generates to the public health system. This way, the anonymized population is grouped, and the effectiveness of this model for management in this organization is assessed.

The rest of the paper is organized as follows. First, the available sources of information will be described. Next, the econometric approach employed will be outlined, followed by the presentation of the main results of the econometric model's fit. Based on this analysis, a capitation distribution will be performed among the different healthcare areas according to the criterion of need. Finally, the study's main conclusions and practical implications will be discussed.

## Materials and methods

### Design and study area

This is a cross-sectional study on public healthcare expenditure in the autonomous community of the Murcia region (South-Eastern Spain, 1.5 million inhabitants) and its application in clinical management using predictive models. In this region, health system is divided into nine health areas, each of which depends on a reference hospital. The database for analysis is derived from information collected by the Analytical Accounting System of the Murcian Health Service (MHS) for the year 2017. Specifically, the data includes disaggregated information for over 1.25 million residents of the Murcia region, including age, sex, AMG, and the total amount of costs attributed to each individual based on the MHS's analytical accounting system for that year. The AMG is a population grouper whose structure considers two factors: multimorbidity and complexity. Thus, based on the diagnostic codes encoded for each person, it classifies the population into exclusive groups based on, on the one hand, their multimorbidity, and simultaneously, the assignment of a complexity value, into different subgroups or levels of complexity. The morbidity groups in which users are classified take into account the typology of diseases (acute, chronic, or oncological), and in the case of the presence of chronic disease, they identify whether it affects a single organ system or more, resulting in the following categories: Healthy population; Pregnancy and/or childbirth; Acute pathology; Chronic disease in 1 system; Chronic disease in 2 or 3 systems; Chronic disease in 4 or more systems;Neoplasms in the period. Each morbidity group (except for the healthy population) is independently divided into 5 subgroups or levels of complexity. This complexity is determined by analyzing different variables of resource utilization such as mortality, risk of hospital admission, visits to primary care, or prescriptions, linked with diagnoses. The complexity calculation was carried out through quali-quantitative models with information on both morbidity and the variables mentioned for the 7.5 million population of Catalonia in the year 2011 (CatSalut data). The 5 subgroups or levels of complexity are obtained by identifying 4 cut-off points from the 40th, 70th, 85th, and 95th percentiles of complexity in each morbidity group of the same population. In this way, we obtain 31 resulting AMG from the combination of morbidity groups and the level of complexity. It serves as a key tool in predicting healthcare needs and optimizing resource allocation, thereby enhancing the efficiency of healthcare delivery within the Spanish healthcare system [10].

### Data sources

The information sources come from two systems used in the field of healthcare management. Firstly, the Centralized Analytical Accounting Information System provides data on the total amount of costs attributed to each individual. Secondly, the Health Card Information System collects data on age, sex, and AMG for each resident of the Murcia region.

The Centralized Analytical Accounting System of the Murcian Health Service (SCAC-SMS) is a centralized cost and activity management system that integrates both Primary and Hospital Care. It provides cost per patient information, with the ultimate goal of understanding and optimizing the costs of the entire organization, sharing and comparing information, managing budgets, enabling clinical management, and facilitating capitation financing. To determine the cost per patient, the process begins with calculating the costs of health areas, centers, services, and the smallest cost allocation unit, the homogeneous functional groups. Subsequently, the cost of different activities and services is calculated. Since these activities and services are provided to various patients, aggregation is used to calculate the cost of patient care.

The SCAC-SMS is founded on a full costing system, which implies that the calculation of costs incorporates all costs generated within the institution, whether direct or indirect, fixed or variable, and related to production, administration, or finance. These costs are fully allocated across all activities, such that any misallocation would result in one activity bearing a higher cost, thereby reducing the cost allocated to another activity by an equivalent amount. Consequently, the total cost volume of the institution for a given fiscal period matches the total amount of expense accounts listed in the SMS's financial accounting information system for the same period, including annual depreciation.

### Econometric model and model assesment

The intended model aims to explain the observed costs from the MHS's Analytical Accounting System based on objective conditions that can account for the healthcare needs of the population. Specifically, the objective conditions are based on age, sex, and AMG.

Where Y_i_ represents the individually observed costs, X_i_ includes the variables capturing the objective conditions (sex, age, AMG) (Eq. 1 in supplementary material).

The simplest way to estimate this model is through Ordinary Least Squares (OLS). However, the nature of healthcare cost data, characterized by high asymmetry, may result in unreliable estimates [22]. An alternative for model estimation is the use of non-linear regression models, among which Generalized Linear Models (GLM) are commonly employed. This approach extends the modeling framework, allowing for non-normally distributed dependent variables. This flexibility has led to frequent use of such models in healthcare cost estimation [23–28].

When selecting the link function and distribution family, we will adopt a dual approach based on information criteria and parametric considerations. The most common link functions are identity, power, and natural logarithm. Distribution families for continuous dependent variables imply that the variance is a power function of the mean with an integer exponent. The most common are the normal distribution, where the variance is constant (null exponent); the Poisson, where variance is proportional to the mean (exponent of one); the gamma, where variance is proportional to the square of the mean (exponent of two); and the inverse Gaussian, where variance is proportional to the cube of the mean (exponent of three).When selecting the link function and distribution family, we will employ a dual approach: one based on information criteria and the other parametric.In the information criteria-based approach, we will estimate the most commonly used GLM models for healthcare costs and use two criteria to make our selection: the Akaike Information Criterion (AIC) and the Bayesian Information Criterion (BIC). The basic idea is to choose the model that minimizes both indicators for the available data, as both measures inform us about the relative quality of a statistical model for a given data set. The main advantage of this approach is that information criteria are not subject to problems arising from multiple hypothesis testing. In the parametric approach, we will sequentially choose the link function and the distribution family. Firstly, we will use the Box-Cox transformation to find the functional form of the link function. The underlying idea is to identify which parameter value, λ, leads to greater symmetry. Secondly, within the parametric approach, we will employ a statistical test based on regression analysis called the Modified Park Test [29]. This test allows us to easily determine the type of relationship between the predicted mean and variance in a generalized linear model. The choice of a distribution family is relevant because it affects the precision of the estimates, although it is true that if the link function is correctly chosen, a mistake in the distribution family selection will not result in inconsistencies in parameter estimates. To conduct the Modified Park Test, we will estimate a model by GLM, which requires choosing initial link function and distribution family. In our case, dealing with healthcare costs, we will use the logarithmic function as the link and the gamma distribution as the family. Importantly, for the test to be correctly conducted, including the correct link function is crucial. Once the model is estimated, we will generate the logarithm of the squared residuals and the linear prediction. Through ordinary least squares regression, we aim to determine the relationship between the first and the second, that is, to estimate the relationship between the error variance and the mean. Thus, the estimated coefficient will indicate which distribution we should choose based on how close it is to the reference values.

### Model specification test, goodness of fit, and model validity

To assess the correctness of model specification, goodness of fit, and model validity, we will conduct various analyses. Model specification will be tested using the Pregibon test [30], the modified Hosmer-Lemesbow test [31], and the Copas test [32]. To approximate goodness of fit, we will use several indicators. Firstly, since models with different error distributions will be employed, we will include the R-squared obtained from an auxiliary regression between observed costs and predicted costs on the untransformed scale, ensuring independence from assumed errors [33]. Additionally, the Root Mean Squared Error (RMSE) and Mean Absolute Error (MAE) will be used. These three indicators will also be calculated for judging goodness of fit using predictions obtained for the Copas test. For GLM estimated models, the explained pseudo-variance through deviance change, equivalent to R-squared for this type of model [34], will also be computed.

### Capitated financing model for the murcian health service

Once the best-fitting model has been determined, a double analysis will be conducted. Firstly, the prediction with the chosen model will be compared with the observed expenditure for each of the health areas into which the Murcian Health Service (MHS) is divided. Moreover, two cross-sectional indices will be provided: one comparing the observed value for each area with the observed value for the entire healthcare system, and another comparing the predicted value for each area with the total. Then, a small simulation exercise will be performed to assess the impact of implementing a capitated financing system in the different areas based on the estimated model. This analysis will take into account the total observed expenditure eligible for distribution according to the analytical accounting of the MHS in that year. The allocation for each area will be calculated based on three criteria. Firstly, a criterion purely of population weight. Secondly, considering the needs of the population, which are incorporated into our model through the adjustment of the population by characteristics of age, sex, and GMA. The population to be used would be the adjusted population, that is, the population adjusted based on the criteria of need. Note that the need index is essentially the cross-sectional index of the predicted values. The third criterion is historical, that is, how the budget has been allocated until now. Finally, the second allocation will be compared with the third.

## Results

### Descriptive analysis

Table 1 shows the characterization of the sample by AMG, Expenditure, Age, and Gender. Based on the accessible data, the mean age is marginally above 40.5 years. Females constitute just over 52.2% of the population, and the average imputed expenditure is slightly more than 1,472 euros. However, significant differences exist in both age and gender when considering different AMG based on health status. Specifically, the youngest average age is found in the group representing acute conditions with a high degree of complexity, just above 9.6 years, while the highest average age is observed in the group with high complexity and chronic pathology in 4 or more systems, surpassing 78.6 years. The percentage of females also varies significantly depending on the considered AMG. For pregnancy-related conditions, it represents 100%, while for the case of the healthy population, the lowest average value is obtained, slightly below 39%. As expected, higher complexity groups are associated with higher average expenditure. Three groups stand out in terms of population weight, exceeding the 10% threshold: group 321 (patients with chronic pathology in 2 or 3 systems and the lowest level of complexity), group 322 (patients with chronic pathology in 2 or 3 systems and the second lowest level of complexity), and group 331 (patients with chronic pathology in 4 or more systems and the lowest level of complexity). Collectively, these three groups account for 417,931 individuals, approximately 33% of the total observations.

**Table 1:** Characterization of the sample by Adjusted Morbidity Groups, expenditure, age, and gender. mean, standard deviation, and number of observations

	Mean			Standard Deviation		Observations
	Age (years)	Expenditure (€)	Women (%)	Age (years)	Expenditure (€)	
AMG						
1	29.75	217.95	38.81	17.84	1649.53	59,406
101	23.96	242.02	40.97	17.35	1416.63	63,069
102	22.30	338.14	42.62	17.83	945.04	40,396
103	18.82	490.77	45.93	17.94	1914.75	28,366
104	14.74	713.83	49.23	17.31	1829.18	17,678
105	9.61	1510.94	51.35	15.44	3692.16	9,398
201	31.31	1323.92	100.00	6.29	1617.92	5,448
202	31.87	2265.04	100.00	6.09	1979.63	8,504
203	32.03	2824.10	100.00	6.11	2054.58	6,473
204	32.23	3526.77	100.00	6.25	2726.36	5,424
205	33.16	4910.26	100.00	6.42	5406.10	2,019
311	27.50	251.55	47.24	15.66	1207.10	51,168
312	31.48	337.31	41.26	18.79	1550.15	85,983
313	31.05	541.05	42.45	19.40	4046.06	60,803
314	30.10	828.60	44.60	21.07	2901.36	46,292
315	28.19	1962.88	44.89	23.25	6029.96	19,558
321	32.89	402.78	52.95	17.48	1304.25	133,841
322	40.78	731.48	51.49	19.57	2420.03	148,624
323	47.53	1221.10	50.68	20.36	3213.40	77,502
324	53.49	2016.29	48.55	20.75	3880.43	44,931
325	61.34	4645.00	40.59	19.86	8752.23	16,830
331	49.91	1173.00	68.30	18.92	3010.10	135,466
332	64.75	2659.36	65.35	15.77	4917.98	96,693
333	70.87	4494.43	59.80	14.09	6789.83	31,732
334	75.04	7046.22	53.81	13.00	10,394.44	23,185
335	78.62	13,135.77	50.34	10.92	15,821.16	11,186
401	56.92	5275.08	52.42	17.11	8030.34	6,984
402	66.53	9960.65	48.43	14.83	12,529.17	6,287
403	70.65	14,769.87	42.88	13.81	17,427.73	3,757
404	73.89	18,873.63	37.26	12.35	22,535.54	2,845
405	76.69	24,280.64	32.62	10.83	23,529.62	1,260
Total	40.57	1472.43	52.22	23.58	4775.14	1,251,108

### Econometric model of healthcare expenditure

Following the information criterion approach, several models were estimated using GLM, choosing different link functions and distribution families that are commonly used in the literature. The results are presented in the Table 2. As observed, following the Akaike criterion, the best models would be those using the gamma distribution family, with no differences based on the chosen link function.

**Table 2:** Selection the link function, distribution family, specification and goodness-of-fit of the estimated models

	AIC			BIC		
Identity Gaussian	19.518			219E + 13		
Logarithmic Gaussian	19.486			2.13E + 13		
Power (0.5) Gaussian	19.503			2.16E + 13		
Identity Inverse Gaussian	19.789			-1.76E + 07		
Logarithmic Inverse Gaussian	19.789			-1.76E + 07		
Power (0.5) Inverse Gaussian	19.789			-1.76E + 07		
Identity Gamma	15.434			-1.57E + 07		
Logarithmic Gamma	15.435			-1.57E + 07		
Power (0.5) Gamma	15.434			-1.57E + 07		
Identity Poisson	1978.709			2.45E + 09		
Logarithmic Poisson	1947.902			2.41E + 09		
Power (0.5) Poisson	1969.199			2.44E + 09		
				IC (95%)		
λ β(Box-Cox)	0.0417			(0.0409,0.0425)		
(Modified Park Test)	18.979			(1.8943,1.9016)		
	Pregibon	Hosmer-Lemesbow	R2	RMSE	MAE	Pseudovariance
MCO	0000	0.000	0.231	4188.547	1233.345	
MCO Ln	0.000	0.000	0.213	4238.790	1205.425	
GLM Power Gamma	0.598	0.000	0.225	4204.572	1202.172	0.440
GLM Logarithmic Gamma	0.403	0.000	0.230	4190.462	1198.393	0.440
GLM Identity Gamma	0.710	0.000	0.225	4203.810	1201.606	0.440
	10 repetitions					
	Copa Test	RMSE	MAE			
MCO	0.002	4190.709	1227.101			
MCO Ln	0.000	4242.258	1197.594			
GLM Power Gamma	0.000	4207.198	1196.252			
GLM Logarithmic Gamma	0.256	4196.172	1190.308			
GLM Identity Gamma	0.000	4205.495	1197.276			

For the parametric approach, first, the λ parameter from the Box-Cox transformation was estimated. As shown in the Table 2, the obtained value is very close to 0. Therefore, we conclude that the highest degree of symmetry is achieved when using the logarithmic link function. Based on this information, it is necessary to perform the Modified Park Test. The test results are presented in the Table 2. It can be observed that the obtained parameter is very close to 2, indicating that the gamma distribution family best captures the relationship between the predicted mean and variance.

Therefore, based on the two approaches conducted, it appears evident that the Gamma distribution performs the best for selecting the distribution family in our GLM model. However, regarding the choice of link function, there is conflicting evidence. On one hand, the information criteria-based approach suggests that the potential or identity link functions marginally fit the available data better, while the sequential approach indicates that a logarithmic function should be employed.

In addition to the GLM models, the estimations using OLS and OLS on logarithmically transformed costs have also been included. As shown in Table 2, only the GLM models pass the Pregibon test. However, the results for the Hosmer-Lemesbow test are not as promising, as none of the estimated models pass this test. Focusing on the adjusted R-squared, we observe that all estimated models achieve very similar fits, with the gamma approach and logarithmic link function achieving the best fit. Regarding the MAE, the smallest value is obtained in the logarithmic approach followed by the potential approach. The RMSE values are similar, although the OLS estimation yields the best quadratic results. As mentioned, the GLM models also estimated the pseudo-variance explained through deviance change, which has a similar interpretation to R-squared in OLS estimation. As observed, all three GLM fits explain a similar portion of the variance.

Based on the conducted analyses, we believe that any of the GLM models could be used for our purposes, but the model with a logarithmic link function appears to be the most suitable. Indeed, the results of the Copas test and the calculated indicators support this notion. As observed, when assessing the predictive capability of the different models using the Copas test, only the logarithmic approach manages to pass the test. Additionally, for all three selected models, this link function achieves both the lowest MAE and the lowest root mean squared error (RMSE).

### Prediction of costs by AMG and by health areas

Table 3 presents the coefficients of the selected model. Additionally, it displays the average marginal effects, which represent the average change in the dependent variable associated with the shift from 0 to 1 in each binary independent variable, averaged across all observations in the sample. This provides a distinct measure of the typical impact of changing a binary independent variable from 0 to 1. Based on these average marginal effects, it can be concluded that the impact of the AMG on total costs is substantially greater than that of age in most instances. This effect is especially significant for AMGs that represent more complex health conditions.^1^

**Table 3:** Coefficients of the selected model, average marginal effects, predicted mean values, and relative weights for the Adjusted Morbidity Groups

	Coeff	Av.Mg.Effect (€)	Predicted (€)	Relative weight
Sex	-0.126	-186.27		
Age group (base = 0)			924.65	
5	-0.509	-742.69	446.42	
10	-0.475	-703.38	464.85	
15	-0.428	-647.75	526.31	
20	-0.429	-648.78	610.51	
25	-0.389	-599.14	725.12	
30	-0.303	-487.01	907.77	
35	-0.276	-448.49	933.75	
40	-0.249	-409.62	960.93	
45	-0.230	-382.05	1108.35	
50	-0.186	-316.02	1407.97	
55	-0.206	-346.39	1686.09	
60	-0.198	-333.45	2119.41	
65	-0.161	-275.98	2721.76	
70	-0.137	-237.81	3427.09	
75	-0.155	-266.69	4087.84	
80	-0.228	-379.26	4492.81	
85	-0.311	-497.51	4550.45	
AMG (base = 1)			215.57	1.000
101	0.095	22.01	239.45	1.111
102	0.416	114.47	336.07	1.559
103	0.761	253.22	488.33	2.265
104	1.085	435.12	710.44	3.296
105	1.762	1070.60	1499.98	6.958
201	1.911	1278.68	1325.84	6.150
202	2.445	2336.80	2273.03	10.544
203	2.665	2966.31	2835.37	13.153
204	2.886	3756.16	3543.37	16.437
205	3.213	5296.28	4951.63	22.970
311	0.198	48.47	252.75	1.172
312	0.445	124.37	337.69	1.566
313	0.908	328.04	542.61	2.517
314	1.308	598.74	826.14	3.832
315	2.122	1629.81	1951.76	9.054
321	0.653	204.43	404.73	1.877
322	1.201	515.29	732.52	3.398
323	1.683	972.45	1225.20	5.684
324	2.169	1719.05	2035.35	9.442
325	2.976	4131.53	4665.86	21.644
331	1.659	944.12	1168.28	5.419
332	2.443	2331.91	2671.43	12.392
333	2.961	4063.36	4512.83	20.934
334	3.406	6467.34	7029.94	32.611
335	4.026	12,216.92	12,991.52	60.266
401	3.131	4858.91	5346.30	24.801
402	3.739	9109.43	10,022.15	46.491
403	4.126	13,525.69	14,816.30	68.730
404	4.360	17,150.43	18,797.00	87.196
405	4.611	22,094.56	24,150.32	112.029
Constant	5.731			

Once the model that best describes our healthcare cost data has been selected, it is possible to predict the average costs associated with each AMG. This information is presented in Table 3. Additionally, the relative weights of each AMG  in comparison to the healthy population group have been calculated. As can be observed, within each morbidity group, the subgroups or levels of complexity demonstrate clearly increasing costs. For instance, for acute illness patients, the first level of complexity only entails an 11% increase in average cost compared to the healthy population group, the second level of complexity results in a 56% increase, and the highest level of complexity represents an almost sevenfold increase in the average cost).

The next step is to predict the average cost that should be observed in each of the different health areas according to this modelling. The analysis focuses on the 9 health areas. The comparison of this prediction with the actually observed values is presented in the Table 4.

**Table 4:** Values and index of average cost per patient observed and predicted by area (Total MHS = 100)

	Values			Index (Total MHS = 100)	
	Observed	Predicted	Difference	Observed	Predicted
Área I	1445.20	1455.45	-10.25	98.11	98.80
Área II	1538.25	1505.97	32.28	104.43	102.23
Área III	1406.21	1385.48	20.73	95.46	94.05
Área IV	1610.49	1499.33	111.16	109.33	101.78
Área V	1425.95	1336.79	89.16	96.80	90.75
Área VI	1369.53	1496.10	-126.57	92.97	101.56
Área VII	1473.27	1527.42	-54.15	100.01	103.69
Área VIII	1553.30	1454.58	98.72	105.45	98.75
Área IX	1678.93	1511.97	166.96	113.98	102.64
Total	1473.07	1473.07	0.00	100.00	100.00

Differences can be observed between the average observed and predicted costs by areas. Specifically, it is noted that for areas I, VI, and VII, the predicted values are higher than the actually observed values. In the case of Area I, the difference amounts to slightly over 10.2 euros per patient, while for Area VII, this difference can be estimated at slightly over 54.1 euros per patient. However, the largest difference is found in Area VI, where it reaches over 126.6 euros per patient. On the other hand, in areas II, III, IV, V, VIII, and IX, the opposite phenomenon is observed, where the observed costs exceed those predicted by the model based on need variables. In Area II, this excess cost can be estimated at just over 32.2 euros; in Area III, the figure amounts to slightly over 20.7 euros; in Area VIII, the value is substantially higher, reaching 98.7 euros; in Area V, the excess is similar to the previous one, around 89.1 euros. The last two areas show the highest excess of costs compared to the model's prediction. In Area IV, this additional cost amounts to around 111.2 euros per patient, while in Area IX, it reaches the maximum absolute value of 167 euros per patient.

Another way to visualize this difference in behaviour between the average observed and predicted costs by areas is by calculating cross-sectional indexes, setting the system average to 100. This information is presented in the Table 4. The fourth column of the table presents the observed situation in terms of average cost per patient in each area relative to the system average. It can be observed that there are areas, such as I or III, whose costs are slightly below the average, while others are clearly above the system average, as is the case in Area IV or Area IX. It is evident that part of these observed differences can be explained based on need criteria, considering the different composition of the covered population, their health needs, etc. The fifth column shows what the average cost per area should be based on the need criteria included in the model (age group, sex, and AMG). As observed, differences still exist, and in some cases, they even increase compared to the observed values, but often they are significantly reduced. In Area I, for example, the observed average cost is slightly below the average, and according to our model, it should be closer to the average than the actually observed cost. A similar situation, but in the opposite direction, is found in Area II. The observed average cost exceeds the system average by 4.4 points, while based solely on need criteria, it should only be 2.2 points above. In Area III, on the other hand, we know that the observed average cost per patient is 4.5 points below the average, but according to the estimated model, it should be nearly 6 points below. A similar pattern is observed in Area IV, but above the reference value. The observed cost exceeds the average by more than 9.3 points, but according to the model's prediction, it should only be 1.8 points above. The comparison for Area V shows that instead of the slightly over 3.2 points below the average that the cost per patient is, it should increase to almost 9.3 points below if we consider the need criteria. However, the opposite phenomenon occurs in Area VI, where instead of the nearly 7 points below the average that the observed cost is, the predicted cost should exceed the average by almost 1.5 points. In Area VII, where the observed cost slightly exceeds the system average, according to the prediction based on need, this difference should widen to almost 3.6 points. Regarding Area VIII, a more extreme behaviour is found, as instead of being above the average by 5.5 points, as observed, the prediction is below the average by around 1.2 points. Finally, Area IX, which has the highest average cost per patient, nearly 14 points above the average, should only be slightly more than 2.6 points above based on need criteria alone.

To illustrate the consequences of implementing a capitation financing system based on healthcare expenditure needs by areas using the estimated model, the following table has been constructed. It simulates the distributional effects by areas of a fixed budget, specifically, 1842 million euros. The choice of this amount is not arbitrary, as it approximates the figure recorded in the MHS analytical accounting for the latest available year, 2017.

To explain the content of the simulation, it is best to provide an example (Table 5). Distributing the total budget among health areas based solely on the covered population would result in, for example, Area I receiving around 329 million euros. However, if we take into account the population's needs as included in our model, the population to consider would be the "adjusted population," which means the population adjusted based on need criteria. Instead of considering that the area has 269,627 people for distribution, which is derived from the health cards, it should be considered that this area covers 266,402 "equivalent persons." Therefore, the volume of resources that should be allocated to finance Area I should amount to approximately 325 million. This figure contrasts with the 317 million that would result from applying the distribution as observed so far, theoretically, based on the observed average cost. In summary, Area I should receive slightly over 7.64 million euros in addition to what it currently receives if a capitation system were effectively used to finance the areas.

**Table 5:** Simulation of the impact of a capitation financing system by health areas for a budget of 1842 million euros. (Persons, millions, euros)

	Covered Population (A)	Need Index (B)	Adjusted Population (A) x (B)	Distribution by areas (€ mill.)			
				Based on Population (C)	Based on adjusted-population(D)	Based on historical cost (E)	Difference (D)—(E)
Area I	269,627	0.98804	266,402	329	325	317	7.44
Area II	288,536	1.02234	294,981	352	360	365	-5.02
Area III	180,577	0.94054	169,840	220	207	210	-2.62
Area IV	69,947	1.01783	71,194	85	87	102	-15.28
Area V	60,828	0.90749	55,201	74	67	74	-6.96
Area VI	272,042	1.01564	276,296	332	337	294	42.76
Area VII	204,969	1.03690	212,532	250	259	260	-0.88
Area VIII	109,851	0.98745	108,473	134	132	139	-6.87
Area IX	54,874	1.02641	56,323	67	69	81	-12.59

Only Area I and Area VI should receive a higher relative funding than what is currently deduced from the analytical accounting. In the case of the latter, it would correspond to an additional 42.7 million euros compared to the distribution based on historical costs (an additional 14.5% of resources). On the contrary, the rest of the areas would be "overfunded" to the extent that their expenditure levels exceed the theoretical ones resulting from allocating expenditure based on objective need criteria (age, sex, and morbidity group). This "excess funding" would range from 0.9 million euros in Area VII to almost 15.3 million euros in Area IV. In relative terms, this downward adjustment would reach a maximum of almost 15.5% of the budget in Area IX and a minimum of 0.3% for Area VII, where the distribution of the budget based on historical costs practically coincides with the resulting distribution using the population adjusted according to need as the allocation criterion.

## Discussion

The present study has contributed to the literature on healthcare financing models by taking into account not only demographic variables such as sex and age but also the health status of the population (AMG). Evidence has been found that AMGs, as population aggregators, can be used as the basis for a needs-based capitation financing system. This evidence reinforces the role that AMGs can play within the public healthcare system in Spain, especially considering that this aggregator is chosen by the Ministry of Health in Spain [35].

This study is part of a growing literature on the superior performance of AMGs, not only in isolation but also when compared to other indicators. For example, there is evidence of the effectiveness of aggregators based on CRGs (Clinical Risk Groups) and AMGs in Spain [36–40]. Although some criticisms have been raised about the design of the AMG-based aggregator, particularly regarding the use of qualitative-quantitative models and even the econometric models employed due to the absence of adjustment measures [41], there are numerous published studies in recent years that support its good performance as a clinical management tool and proactive approach to health policies. For instance, it was compared the predictive capacity for high-complexity patients of the two most commonly used tools, CRGs and AMGs, in 18 health zones of the Canary Islands during the period 2013–2014 [42]. According to the study, AMGs outperform CRGs in predicting hospital admissions.

In the Community of Madrid for the period 2015–2016 was found that the level of utilization of primary care services for chronic patients increased with the level of risk assigned by the AMG, highlighting the usefulness of AMGs for clinical and healthcare management [43]. This aspect is particularly relevant considering that almost 80% of primary care consultations, around 60% of hospital admissions, and 33% of emergency visits can be attributed to chronic patients [44].

Several studies have been carried out in Catalonia [38, 45–47]. For example, it was conducted a clinical validation of the two morbidity aggregators (AMG and CRG) in the primary care setting in Catalonia [38]. For this purpose, 40 primary care physicians were paired and assessed 25 different medical records per pair. The study found that there was greater agreement between evaluators for AMGs than for CRGs (0.63 vs. 0.35). Additionally, the physicians were asked to rate the goodness of both aggregators, and the ratings were very similar for both. However, for profiles of higher complexity, AMGs received better scores. According to the authors, this study is the first clinical validation study of morbidity aggregators and states that "AMGs present a morbidity stratification comparable to CRGs but use less information (do not include procedures, pharmaceutical prescription, patient age, or diagnostic scope), and in higher-risk strata, they perform better." In the same line, it was compared the explanatory capacity of AMGs in predicting health outcomes with other quantitative measures of multimorbidity using data from 2017 [45]. Specifically, these authors focused on 6.2 million patients retrospectively and employed five multimorbidity measures: Charlson Index Score, count of chronic diseases following different proposals (“Quality and Outcome Framework of the NHS, healthcare cost and utilization project [HCUP] of the US Agency for Healthcare Research and Quality”, and the proposal of the Karolinska Institute), and AMG classification. They used diverse health outcomes measures (e.g., death, hospitalization, unplanned hospitalization, primary care and specialist visits, medication use, nursing treatment admission, and high cost) and analysed the results by population subgroups. According to the study, AMGs performed the best and showed the greatest consistency in all population groups. Another similar study analysed the validity of AMGs compared to CRGs in the primary care setting using data from Catalonia for the year 2014 [46]. The study focused on the goodness of fit and explanatory power of both stratification tools for three indicators: urgent hospital admission, number of visits, and pharmacy expenditure. According to their data, the fit using a generalized linear model including sex, age, and morbidity group was superior for AMGs than for CRGs, leading them to conclude that AMGs are a useful tool for measuring the burden of morbidity in primary care. Finally, using data for the year 2015, it was compared the predictive capacity of the Charlson Index, the number of chronic diseases, and two population stratification measures (AMG and CRG) with respect to four variables of interest for primary care: more than 12 visits to primary care in a year, home care, use of social workers, and polypharmacy [47]. For the first three variables, the predictive capacity of AMGs was superior to that of CRGs, although the result was reversed for the fourth variable.

Similar results were obtained in Aragón, Castilla y León, and the Canary Islands [48]. These authors compared the predictive power of AMGs with two other population stratification tools: ACGs and CRGs. The data covered the period 2014–2016 for. Specifically, they measured the predictive power with respect to the probability of death, the probability of having an urgent hospital admission, the number of visits to hospital emergency departments, the number of primary care visits, the total number of outpatient consultations, and pharmaceutical expenditure. The conclusion they reached was that the predictive capacity of AMGs is at least as good as that of the other two indicators.

From our perspective, our work constitutes a substantial advance over the existing literature to date. On one hand, all costs used come from the analytical accounting system of the MHS, which is not common practice, given that it is generally customary to use costs derived from public rates or tariffs (Public Tariffs Law) [12, 36, 39], which are merely an approximation to the true cost. On the other hand, our work employs a very broad population base, referring to the entire regional health system and for all costs, not just one or several health areas as is usually the case [36, 39], or only referring to a type of costs such as pharmaceuticals [42, 48]. Finally, our work represents a feasibility analysis for the implementation of a capitation financing system for budget allocation among the different health areas of a Spanish region. To the best of our knowledge, this is the first work that analyzes this paradigm shift in public health financing in Spain with population-based data and referring to the entire system. So far, attempts to establish this type of budgetary allocation in Spain have been very limited, only to the hospital setting [49], despite there having been multiple approaches in OECD countries in this direction in recent years [50–52]. The latest evidence available seems to suggest that the establishment of these systems for financing can lead not only to efficiency gains but also in terms of improving healthcare and avoiding complications in the hospital setting [53], although it could entail a certain centralization of more complex surgical procedures [54].

## Limitations

This study is not without limitations. The main limitation lies in the nature of the data itself. Specifically, the analytical accounting system only records users who actually incurred expenses within the system during 2017. This limitation arises from the fact that it would have been desirable to have information about citizens who, despite having the right to healthcare, did not generate any expenses within the system. If data were available for patients who have not incurred any costs to the system, the econometric approach would have been different, as it would have been possible to employ a two-part model. However, we believe that this limitation is partially addressed by the breadth and representativeness of the available database. On the other hand, the analytical accounting system of the MHS (Health Service) does not allocate a significant portion of the expenses, exceeding 10%, to individual patients. This is because certain items, such as the 061 emergency service or central services, either lack sufficient information for allocation, lack reliable allocation criteria, or have not been deemed appropriate for allocation at this time. To the best of our knowledge, this limitation will diminish over time, as the MHS administration is firmly committed to improving the system and expanding the allocation of expenses to each patient. Nevertheless, it is evident that this constitutes a limitation of the study since a portion of the expenses could not be analysed. Another limitation of this study is that the proposed implementation of the capitation financing system is not fully developed. However, we consider this criticism, although valid, to be relative, as our ultimate objective is to foster debate and progress towards the development of a financing system that promotes greater territorial equity while facilitating a more efficient use of public resources dedicated to healthcare in our region through "competition by comparison" [55].

## Conclusions

Estimating a model for public healthcare expenditures based on expenditure needs associated with the composition of sex, age, and morbidity represented by the AMG has allowed us to identify differences in these needs across health areas within the MHS. In line with our pure capitation financing system, there are health areas whose observed costs are substantially lower than the predicted costs, indicating clear underfunding. On the other hand, there are areas that are spending more than estimated to meet the needs of their patients, indicating overfunding. Further research is needed to examine plausible ways to adjust the funding of these areas to correct these differences.

### Supplementary Information

Supplementary Material 1.

## Data Availability

The data that support the findings of this study are available from Murcian Health Service (SMS), but restrictions apply to the availability of these data, which were used under licence for the current study and so are not publicly available. The data are, however, available from the authors upon reasonable request and with the permission of Murcian Health Service (SMS).

## Footnotes

1 Regarding the error of the predictions, approximated through the MAE, it should be noted that the error increases with age and also escalates with the complexity of the AMG.
