# Supplementary material for: Predicting healthcare expenditure based on Adjusted Morbidity Groups to implement a needs-based capitation financing system
Source: Health Econ Rev. 2024 May 8;14:33. doi: 10.1186/s13561-024-00508-4 (PMC11077809; doi:10.1186/s13561-024-00508-4)
Supplement: Supplementary file 1 — Supplementary Material 1. [file 13561_2024_508_MOESM1_ESM.docx]

EQUATION 1

$$Y_{i}=\beta X_{i}+\epsilon_{i}$$
